# Improving Quality of Care in Rheumatoid Arthritis Through Mobile Patient-Reported Outcome Measurement: Focus Group Study

**DOI:** 10.2196/15158

**Published:** 2020-05-27

**Authors:** Sonali Desai, Emma Stevens, Srinivas Emani, Peter Meyers, Maura Iversen, Daniel H Solomon

**Affiliations:** 1 Brigham & Women's Hospital Boston, MA United States; 2 Division of General Medicine, Department of Medicine Brigham & Women's Hospital Boston, MA United States; 3 Partners Healthcare Boston, MA United States; 4 Department of Physical Therapy, Movement & Rehabilitation Services Bouve College of Health Sciences Northeastern University Boston, MA United States; 5 Division of Rheumatology Brigham & Women's Hospital Boston, MA United States

**Keywords:** quality improvement, rheumatoid arthritis

## Abstract

**Background:**

Patient-reported outcomes (PROs) for chronic disease management can be integrated into the routine workflow by leveraging mobile technology.

**Objective:**

The objective of our study was to describe the process of our quality improvement (QI) efforts using tablets for PRO collection in a busy, academic rheumatology practice to support a treat-to-target (TTT) approach for rheumatoid arthritis (RA) management.

**Methods:**

Our QI team designed a process for routine collection of PROs for RA patients at the Arthritis Center, employing information technology and an electronic medical record (EMR) system. Patients received a tablet at the clinic check-in desk to complete the Routine Assessment of Patient Index Data 3 (RAPID3) survey, a validated RA PRO. RAPID3 scores were uploaded to the EMR in real time and available for use in shared decision making during routine office visits. Weekly data were collected on RAPID3 completion rates and shared with front desk staff and medical assistants to drive improvement. Patients in our patient family advisory council and focus groups provided informal feedback on the process.

**Results:**

From May 1, 2017, to January 31, 2019, a total of 4233 RAPID3 surveys were completed by 1691 patients. The mean age of patients was 63 (SD 14) years; 84.00% (1420/1691) of the patients were female, and 83.00% (1403/1691) of the patients were white. The rates of RAPID3 completion increased from 14.3% (58/405) in May 2017 to 68.00% (254/376) in September 2017 and were sustained over time through January 2019. Informal feedback from patients was positive and negative, relating to the usability of the tablet and the way rheumatologists used and explained the RAPID3 data in shared decision making during the office visit.

**Conclusions:**

We designed a sustainable and reliable process for collecting PROs from patients with RA in the waiting room and integrated these data through the EMR during office visits.

## Introduction

### Background

Patient perspectives must be incorporated into chronic disease management to improve the quality of care. Patient-centered care integrates patient preferences and values into clinical decisions, and according to the Institute of Medicine, it is one of the six pillars of quality health care [1]. However, there remains a gap in how patient values and preferences are integrated into busy clinical workflows. Patient-reported outcomes (PROs) represent validated tools providing patients’ perceptions of well-being and functional status [2]. PROs have been used successfully in clinical settings where the intervention can often lead to a significant change in outcome, such as oncology and joint replacement surgery [3].

Rheumatoid arthritis (RA) can lead to joint destruction and impaired functional status. Treat-to-target (TTT) approach serves as the prevailing treatment strategy for RA management, requiring clinicians to measure disease activity regularly and use these data to guide medication changes, with a goal of achieving remission or low disease activity [4-6]. In Sweden, a national rheumatology quality registry captures 85% of patients with RA and incorporates PRO in routine RA care, using a dashboard to allow patient participation and engagement in RA disease management [7]. PROs offer the promise of increasing value, improving efficiency, and enhancing shared decision making for chronic disease management; however, PROs must be implemented in a thoughtful manner that fits within busy clinical workflows [8].

The Routine Assessment of Patient Index Data 3 (RAPID3) is an RA PRO widely used in RA that has been validated and recommended as a reliable measure of disease activity and can be completed in less than 1 min by patients using a paper form [9-12]. The RAPID3 questionnaire consists of 3 sections: a physical function assessment, a global assessment for pain, and a global assessment of overall health. The RAPID3 questionnaire exclusively relies on patient-derived assessments and does not require input from the clinician.

### Objectives

We describe our initial quality improvement (QI) process leveraging mobile technology by using tablets to collect the RAPID3 disease activity scores from RA patients before office visits. The RAPID3 data are immediately available to rheumatologists in the electronic medical record (EMR) and can be used for shared decision making between rheumatologists and patients. We assessed patient satisfaction on the usability of tablets and their perceptions of how RAPID3 data were used within the clinical visit.

## Methods

### Setting

We conducted our QI work at an outpatient rheumatology practice within a large academic medical center, which uses a vendor-based EMR (Epic) and a patient portal for EMR access. Approval from the Partners Institutional Review Board was waived as this study was part of a larger QI initiative. There were 55 rheumatology attendings and fellows who see RA patients at the Arthritis Center with full-time and part-time schedules and 5 medical assistants. Front desk staff were shared with our adjacent orthopedics outpatient practice and radiology outpatient suites. There were approximately 500 rheumatology patients seen per week, with 15.0% (75/500) having a diagnosis of RA and thus eligible for a tablet to complete the RAPID3 PRO questionnaire.

### Software Development and Information Technology Support

The Arthritis Center purchased tablet devices for PRO collection. Tablets were approved and encrypted by our health care delivery system information technology and PRO team. Tablets were placed in a protective case for storage, charged nightly, and kept on a cart behind the front desk. Staff sanitized the tablets between each use and at the beginning and end of each day. The health care delivery system information technology and PRO team provided support for (1) customizing the tablets with the RAPID3 questionnaire, (2) programming International Statistical Classification of Diseases-Tenth Revision (ICD-10) codes for RA into the tablets, (3) repairing and replacing tablets, and (4) conducting staff training pertaining to the use of tablets in the clinic. The PRO team customized the initial programming and data displays for the tablets based on input from the QI team.

### Patient Perspective

We obtained patients’ perspective on the tablet questionnaire completion and the use of RAPID data in discussions with the rheumatologist during the office visit informally. We conducted seven focus groups as part of our broader QI initiative, 3 focusing on general experience of RA patients and four focusing on shared decision making [13]. We also created an RA patient family advisory council (PFAC), which is a group of patients who are invited to serve as members to help provide patient feedback and input on various RA clinic processes. During our focus groups and our regularly scheduled RA PFAC meeting, we asked patients about their experiences with the RAPID3 questionnaire completion in the waiting room and their perceptions of the use of RAPID3 during office visits.

### Quality Improvement and Process Redesign

This pilot for using tablets to collect RAPID3 PROs was a part of a larger QI initiative designed to integrate a TTT approach for the management of RA into the outpatient rheumatology practice. A team of stakeholders, including rheumatologists, nurses, pharmacists, medical assistants, front desk staff, practice leadership (managers and medical director), project coordinators, and QI leadership, met to develop the pilot program. We assessed patients’ perspective regarding the use of tablets for the collection of PROs through informal qualitative feedback from patient focus groups and our RA PFAC [13].

Multiple small group meetings were held, and a series of small plan-do-study-act (PDSA) cycles took place to develop a workflow that was effective for reliable and sustainable PRO collection. In PDSA cycle 1, front desk staff were given instructions on how to provide RA patients with tablets; however, given the lack of a clear reminder, patients were often missed. In PDSA cycle 2, a project coordinator created a daily list of all RA patients with appointments and gave this list to the front desk staff. As the daily list was not used in the busy front desk staff workflow, patients continued to be missed. In PDSA cycle 3, front desk staff were trained on how to identify eligible RA patients through the schedule view in the EMR; however, we found that not all front desk staff could reliably use this visual cue when there were multiple patients checking in for appointments, and patients continued to be missed.

In PDSA cycle 4, we convened the administrative leadership of the clinic and the front desk staff together to meet with our QI team and discuss barriers and workflows and redesigned the workflow. We found that if patients received the tablet but were brought from the waiting room to the examination room for their visit, they did not always have enough time to complete the RAPID3 questionnaire, leading to a higher volume of questionnaires that were started but not completed. We educated our medical assistants, the staff who bring patients from the waiting room to the examination room to do vital signs, to remind patients to complete the questionnaires. We also started sending weekly emails with the rates of RAPID3 completion to the front desk staff and administrative leadership. With the combination of leadership support and accountability and a better understanding of front desk workflows, we found that by month 4 of our pilot, rates of completion had increased substantially.

### Workflow and Process

All rheumatology patients presented to a central front desk area that was shared by the rheumatology, radiology, and orthopedic departments. Patients with a diagnosis of RA were identified through the initial programming of the tablets via ICD-10 codes, and an icon was displayed on the EMR scheduling interface used by the front desk staff, denoting that a particular patient should receive a tablet during their check-in process.

**Figure 1:**
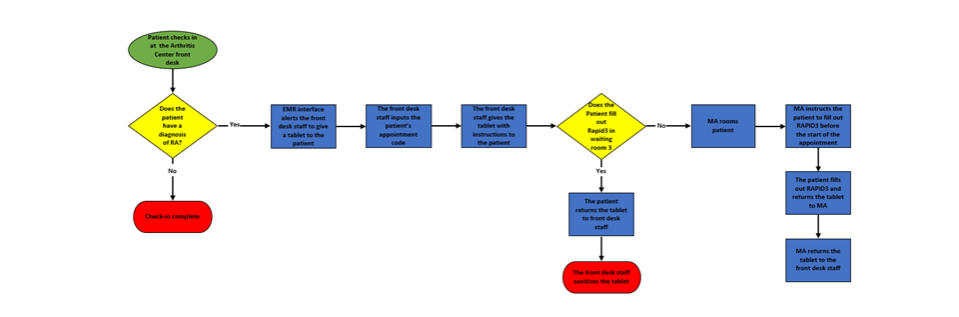
Process flow map of patients and Arthritis Center staff for RAPID3 completion on tablets in waiting room. This figure delineates the process from when patients present to the front desk for their rheumatology office visit to completion of the Routine Assessment of Patient Index Data 3 patient-reported outcome either in the waiting room or in the examination room. EMR: electronic medical record; MA: medical assistant; RA: rheumatoid arthritis; RAPID: Routine Assessment of Patient Index Data.

The front desk staff entered patients’ appointment codes into the tablet and provided patients with the tablet along with instructions to fill out the RAPID3 questionnaire while in the waiting room. A medical assistant was responsible for bringing patients from the waiting room to the examination room. If patients did not complete the RAPID3 questionnaire before the medical assistant called them for their appointment, patients were instructed to bring the tablet into the examination room and complete the questions before the rheumatologist entered the room (see Figure 1). Used tablets were given to the medical assistant, who then returned the tablets to the front desk staff.

### Pilot Study

In May 2017, we began piloting the use of tablets for the RA PRO RAPID3 questionnaires for all RA patients in our Arthritis Center. Weekly emails were sent out to the front desk staff and practice leadership identifying the total number of eligible patients who should have received the tablet but did not, patients who received the tablet but did not complete the RAPID3 questionnaire in its entirety, and patients who received and fully completed the RAPID3 questionnaire. Selected rheumatologists participated in a QI initiative that included attending monthly meetings to promote engagement in a TTT approach for RA through education and training on the use of RAPID3 for RA patients.

### Data Analysis

We present proportions and means of sociodemographic characteristics of patients who completed the RAPID3 questionnaire. We used the SPSS version 24 software for the analysis.

## Results

Between May 1, 2017, and January 31, 2019, a total of 4233 RAPID3 surveys were completed by 1691 patients. Table 1 shows the sociodemographic characteristics of patients who completed the RAPID3 questionnaire. We found that 82.70% (1398/1691) of patients were female, with a mean age of 61.6 (SD 14.4) years, 82.30% (1391/1691) were white, 60.00% (1014/1691) reported that they were married or in a civil union, and 54.70% (890/1691) had a college degree or higher level of education, and 45.30% (766/1691) of the patients who completed the questionnaire did not have a college degree.

**Figure 2:**
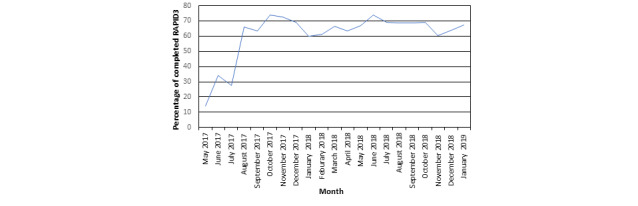
Routine Assessment of Patient Index Data 3 rheumatoid arthritis patient-reported outcomes percent completion rate from May 2017 to January 2019. This figure shows the rate of Routine Assessment of Patient Index Data questionnaire completion 14.3% (58/405) in May 2017 to 67.5% (254/ 376) in January 2019, with a sustained rate of greater than 63.3% (210/331) after September 2017. RAPID3: Routine Assessment of Patient Index Data.

During the study, the overall monthly completion rate of RAPID3 PROs improved, increasing from 14.3% (58/405) in May 2017 to 67.5% (254/ 376) in January 2019, with a sustained rate of greater than 63.3% (210/331) after September 2017 (Figure 2). Informal feedback from patients in our focus groups and our PFAC was both positive and negative: positive feedback centered around the ease of use of the tablet-based PRO and short duration needed to complete the RAPID3 questionnaire; however, patients perceived that rheumatologists did not use RAPID3 data during office visits.

**Table 1:** Characteristics of rheumatoid arthritis patients who completed the Routine Assessment of Patient Index Data 3 questionnaire (May 2017 to January 2019; N=1691).

Patient characteristics	Values
Female, n (%)	1398 (82.70)
Age (years), mean (SD)	61.8 (14.40)
Race (white American), n (%)	1385 (82.30)
Marital status (married or/civil union), n (%)	1014 (60.00)
Education level (graduated from college, graduated from school, or postgraduate), n (%)	890 (54.70)

## Discussion

### Principal Findings

These pilot data illustrate that the use of tablets to collect RA PROs in real time while patients are awaiting their rheumatology appointment can be integrated successfully into the clinic workflow. We demonstrated that these efforts can be sustainable through the use of a multi-stakeholder collaboration between our Arthritis Center staff, our information technology staff, PROs teams, and our QI team. We used an iterative, continuous QI approach with PDSA cycles to modify our process over time.

RAPID3 data reporting was a challenge at times because of changes in the governance within and developing infrastructure of our PROs team. For example, calculating weekly completion rates of RAPID3 questionnaires was conducted through data extraction from a centralized reporting dashboard by our QI team. However, to measure RAPID3 scores, as the centralized dashboard had as yet to offer data export, we had to conduct manual chart reviews in the EMR and link this with a data extract obtained manually from our IT PROs team. Moving forward, the PROs team has created a new centralized data infrastructure to support local ambulatory practices and to support teams extracting customized data and reports to drive improvement efforts. Incorporating PROs within the clinic visit presents a major challenge in a busy ambulatory clinic setting. In other conditions where PROs are collected, questionnaires may be distributed to all patients to facilitate the ease of data collection. Electronic PROs have also been implemented system wide with more general health-related quality of life questionnaires but require strong senior administrative leadership support and local clinic buy-in for success [14]. In our study, we provided the RAPID3 questionnaire only to RA patients. However, patients present with competing clinical priorities during routine follow-up visits; therefore, reviewing and discussing the RAPID3 data may not always be a shared priority for patients and rheumatologists. Within focus groups, some patients reported that the rheumatologist might state the RAPID3 score, but the patient did not know what the score meant and thus could not understand how the RAPID3 score was being used in the clinical discussion.

Much of the value in PROs in clinic comes from assessing symptom severity, informing treatment decisions, tracking outcomes, and prioritizing patient-provider discussions [15]. As the RAPID3 questionnaire measures global patient pain and functional status for RA patients, the completed answers on the questionnaire are readily available to rheumatologists during the office visit and can promote shared decision making when patients are not doing well. The RAPID3 data can be trended over time graphically, allowing the rheumatologist to show these data to the patient during the office visit. The drawback of the RAPID3 questionnaire is that the pain score may be driven by non-RA–related pain; this can confuse patients and clinicians when considering whether treatments require changing.

### Strengths and Limitations

**Figure 3:**
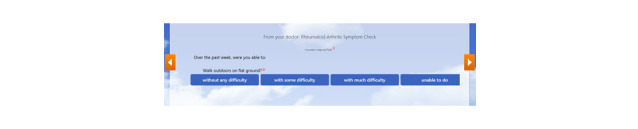
Screenshot of the Routine Assessment of Patient Index Data 3 patient-reported outcome questionnaire. This figure is a depiction of what the patient sees when they are completing the Routine Assessment of Patient Index Data 3 questionnaire on the tablet with a single question per screen in an easy-to-use display.

The use of the RAPID3 tool itself has both strengths and limitations. Key strengths are the ease and speed to complete the RAPID3 questionnaire without medical professional supervision, and the lack of additional components to calculate the score such as laboratory results or joint counts (Figure 3). In particular, our project shows that the RAPID3 questionnaire can be completed by patients with varying educational levels because we found that less than half of our patients who completed the questionnaire did not have a college degree.

Limitations include the fact that the RAPID3 global pain score is generic, so pain that is attributable to etiologies other than RA may hyperinflate the RAPID3 score; this issue can negatively impact the buy-in from rheumatologists to use the data to inform shared decision making for RA management. However, we recognize that patient engagement in shared decision making is an important aspect of medical care delivery and patient satisfaction with medical care, and PROs can help achieve this [16]. Literacy may also impact the completion of a PRO.

### Conclusions

We plan to expand the use of RAPID3 as an RA PRO across all of our rheumatologists and to integrate shared decision making into our daily practice of RA management following the TTT strategy. By reviewing RAPID3 scores with patients during office visits, discussing their meaning in relation to RA and other medical conditions, and integrating the routine measurement of disease activity into RA management, we can increase the use of a TTT approach for improving the quality of RA care.

## Abbreviations

EMR: electronic medical record
ICD-10: International Statistical Classification of Diseases-Tenth Revision
PDSA: plan-do-study-act
PFAC: patient family advisory council
PRO: patient-reported outcome
QI: quality improvement
RA: rheumatoid arthritis
RAPID: Routine Assessment of Patient Index Data
TTT: treat-to-target

## References

[1] Institute of Medicine, Committee on Quality of Health Care in America (2001). Crossing the Quality Chasm: A New Health System for the 21st Century.

[2] Weldring T, Smith SM (2013). Patient-Reported Outcomes (PROs) and Patient-Reported Outcome Measures (PROMs). Health Serv Insights.

[3] Rotenstein LS, Huckman RS, Wagle NW (2017). Making patients and doctors happier - the potential of patient-reported outcomes. N Engl J Med.

[4] Smolen J, Breedveld FC, Burmester GR, Bykerk V, Dougados M, Emery P, Kvien TK, Navarro-Compán MV, Oliver S, Schoels M, Scholte-Voshaar M, Stamm T, Stoffer M, Takeuchi T, Aletaha D, Andreu JL, Aringer M, Bergman M, Betteridge N, Bijlsma H, Burkhardt H, Cardiel M, Combe B, Durez P, Fonseca JE, Gibofsky A, Gomez-Reino JJ, Graninger W, Hannonen P, Haraoui B, Kouloumas M, Landewe R, Martin-Mola E, Nash P, Ostergaard M, Östör A, Richards P, Sokka-Isler T, Thorne C, Tzioufas AG, van Vollenhoven R, de Wit M, van der Heijde D (2016). Treating rheumatoid arthritis to target: 2014 update of the recommendations of an international task force. Ann Rheum Dis.

[5] Huizinga T, Knevel R (2015). Rheumatoid arthritis: 2014 treat-to-target RA recommendations--strategy is key. Nat Rev Rheumatol.

[6] Stoffer MA, Schoels MM, Smolen JS, Aletaha D, Breedveld FC, Burmester G, Bykerk V, Dougados M, Emery P, Haraoui B, Gomez-Reino J, Kvien TK, Nash P, Navarro-Compán V, Scholte-Voshaar M, van Vollenhoven R, van der Heijde D, Stamm TA (2016). Evidence for treating rheumatoid arthritis to target: results of a systematic literature search update. Ann Rheum Dis.

[7] Nelson EC, Eftimovska E, Lind C, Hager A, Wasson JH, Lindblad S (2015). Patient reported outcome measures in practice. Br Med J.

[8] Wagle NW (2017). Implementing patient-reported outcome measures. NEJM Catal.

[9] Pincus T, Swearingen CJ, Bergman M, Yazici Y (2008). RAPID3 (Routine Assessment of Patient Index Data 3), a rheumatoid arthritis index without formal joint counts for routine care: proposed severity categories compared to disease activity score and clinical disease activity index categories. J Rheumatol.

[10] Yazdany J, Robbins M, Schmajuk G, Desai S, Lacaille D, Neogi T, Singh JA, Genovese M, Myslinski R, Fisk N, Francisco M, Newman E (2016). Development of the American College of Rheumatology's Rheumatoid Arthritis Electronic Clinical Quality Measures. Arthritis Care Res (Hoboken).

[11] Pincus T, Swearingen CJ, Bergman MJ, Colglazier CL, Kaell AT, Kunath AM, Siegel EL, Yazici Y (2010). RAPID3 (Routine Assessment of Patient Index Data) on an MDHAQ (Multidimensional Health Assessment Questionnaire): agreement with DAS28 (Disease Activity Score) and CDAI (Clinical Disease Activity Index) activity categories, scored in five versus more than ninety seconds. Arthritis Care Res (Hoboken).

[12] Hendrikx J, de Jonge MJ, Fransen J, Kievit W, van Riel PL (2016). Systematic review of patient-reported outcome measures (PROMs) for assessing disease activity in rheumatoid arthritis. RMD Open.

[13] Forman M, Leatherwood C, Iversen MD, Solomon DH, Desai SP (2019). Quality improvement for rheumatoid arthritis care: results from a focus group. Clin Exp Rheumatol.

[14] Biber J, Ose D, Reese J, Gardiner A, Facelli J, Spuhl J, Brodke D, Lee VS, Hess R, Weeks H (2017). Patient reported outcomes - experiences with implementation in a University Health Care setting. J Patient Rep Outcomes.

[15] Lavallee DC, Chenok KE, Love RM, Petersen C, Holve E, Segal CD, Franklin PD (2016). Incorporating patient-reported outcomes into health care to engage patients and enhance care. Health Aff (Millwood).

[16] Santana MJ, Feeny D (2014). Framework to assess the effects of using patient-reported outcome measures in chronic care management. Qual Life Res.

